# Stackelberg Game Approach for Service Selection in UAV Networks

**DOI:** 10.3390/s23094220

**Published:** 2023-04-23

**Authors:** Abdessalam Mohammed Hadjkouider, Chaker Abdelaziz Kerrache, Ahmed Korichi, Yesin Sahraoui, Carlos T. Calafate

**Affiliations:** 1 LINATI Laboratory, Department of Computer Science and Information Technology, Kasdi Merbah University of Ouargla, 30000 Ouargla, Algeria; hadjsalim30@gmail.com (A.M.H.);; 2 Laboratoire d’Informatique et de Mathématiques, Université Amar Telidji de Laghouat, 03000 Laghouat, Algeria; ch.kerrache@lagh-univ.dz; 3 Computer Engineering Department (DISCA), Universitat Politècnica de València, 46022 Valencia, Spain

**Keywords:** UAVs, services selection, game theory, Stackelberg, mobile edge computing

## Abstract

Nowadays, mobile devices are expected to perform a growing number of tasks, whose complexity is also increasing significantly. However, despite great technological improvements in the last decade, such devices still have limitations in terms of processing power and battery lifetime. In this context, mobile edge computing (MEC) emerges as a possible solution to address such limitations, being able to provide on-demand services to the customer, and bringing closer several services published in the cloud with a reduced cost and fewer security concerns. On the other hand, Unmanned Aerial Vehicle (UAV) networking emerged as a paradigm offering flexible services, new ephemeral applications such as safety and disaster management, mobile crowd-sensing, and fast delivery, to name a few. However, to efficiently use these services, discovery and selection strategies must be taken into account. In this context, discovering the services made available by a UAV-MEC network, and selecting the best services among those available in a timely and efficient manner, can become a challenging task. To face these issues, game theory methods have been proposed in the literature that perfectly suit the case of UAV-MEC services by modeling this challenge as a Stackelberg game, and using existing approaches to find the solution for such a game aiming at an efficient services’ discovery and service selection. Hence, the goal of this paper is to propose Stackelberg-game-based solutions for service discovery and selection in the context of UAV-based mobile edge computing. Simulations results conducted using the NS-3 simulator highlight the efficiency of our proposed game in terms of price and QoS metrics.

## 1. Introduction

Cloud computing (CC) is a concept that refers to the remote exploitation of hardware and software resources located in a data center. With the emergence of mobile applications, Mobile Cloud Computing (MCC) [1] is introduced as an integration of CC into the mobile environment. This type of cloud allows access to services that are provided by the cloud computing environment anytime and anywhere from mobile devices, such as smartphones and tablets.

In this context, a cloudlet is a small-scale cloud computing infrastructure that is located at the edge of the network, closer to end users. It is designed to provide low-latency access to computing resources for mobile and wireless devices. A cloudlet can be thought of as a mini data center that is deployed at the edge of the network, typically within a few miles of the end-user [2].

However, in recent years, a new trend in computing is happening with the function of Clouds being increasingly moving toward the user’s edges, mobile edge computing (MECs) have limited computational capacity but are located close to users. Each MEC may serve multiple users facing a high number of computation tasks, huge application demands, and offering more and better performance benefits [3]. In particular, the use of MEC is preferred to improve communication and processing by: (i) offering a real-time interaction between the MEC server and end devices, (ii) reducing latency of the energy consumption of end devices, communication, and computation, and (iii) decreasing bandwidth because data processing is closer to the source for apps that generate many data, hence contributing to minimizing network data transmission [4].

Recently, we could witness an increasing adoption of unmanned aerial vehicles (UAVs) in a variety of domains, including military, surveillance, telecommunications, monitoring, rescue operations, wildfire management, medical supply delivery, and many other tasks [5,6]. UAVs have the potential to perform various essential tasks in an automated, cheap, and efficient way. UAVs can be controlled manually by a pilot on the ground, or autonomously by a computer program. A typical UAV is made up of a frame, motors, control unit, onboard sensors, communication system, and power supply. Furthermore, many UAVs have a dual tube substructure to allow for the installation of various payloads [7].

If we combine the two concepts defined above, UAV clouds represent a particular class of MCC that combines the concept of CC and UAVs. However, different from CC, whose infrastructure offers virtually limitless resources, but is located far from users, hence introducing delays and bandwidth restrictions, UAVs are very close to users, which can bring numerous advantages. For instance, the UAV may act as a service provider by offering various services to users in the MEC server, such as monitoring, search and rescue operations, surveillance, and delivery of goods.

UAV-enabled MEC networks have the potential to offer high performance and affordable services to end-users, leveraging their deployment flexibility even in rough terrains or deserts, where the terrestrial MEC networks may be impractical, in addition to their short latency, which can be further decreased by optimizing the flight trajectory of the UAVs.

Game theory is a study of mathematical models, and in particular interactive decision-making, where the outcome of each participant or “player” depends on the actions of all [8], is of interest in this work. In addition, Stackelberg is one of the game theory models that can be adopted to solve some UAV-related problems in smart cities, including traffic monitoring and communication [9]. In this game, one player serves as the leader, while the others act as followers. Therefore, the leader should choose and determine the best strategy, assuming that the followers react rationally to optimize their objective functions in light of their leader’s actions [10]. Hence, in the context of our work, Stackelberg game theory can be applied to select the best service and an adequate provider for an user. As a result, users inherently expect to obtain as many services as possible while accounting for their limited budgets; having a limited amount of services, UAVs aim at maximizing their utility by attracting users to purchase their services. To make use of UAV services, the consumer should be able to discover providers, and to acquire information about the different services offered prior to any actual request. Thus, the user will select the most adequate provider in order to consume the required service, given that each UAV service provider can offer services that differ depending on specific features, limitations, and prices. We find that there are numerous challenges in selecting the best service and a suitable service provider in terms of service cost, duration of execution, and energy consumption.

To address the aforementioned challenges, in this paper we propose a Stackelberg-game-based approach to align users’ interests and UAVs, such that each user is satisfied with its obtained service, while UAVs maintain a considerable revenue. The main contributions of this work are the following:

- We establish the interaction between users and UAV service providers, aiming to select the best service provider in the UAV-based MEC.
- We conduct the simulation of our game with two players to make a performance assessment of our approach.

The remainder of this work is organized as follows: in Section 2 we present related works on this topic. We then introduce system models in Section 3. The problem formulation is discussed in Section 4. We present the Stackelberg game theory approach in UAV-based MEC in Section 5, while in Section 6 we investigate the simulation results obtained, with discussion. Finally, the main conclusions and future research work are presented in Section 7.

## 2. Related Work

The cloud provides enormous resources to facilitate the pre-processing and storage process, especially in the medical field [11], as algorithms based on artificial intelligence, such as fuzzy intelligence learning approach [12], take advantage of many of these resources in order to accelerate feature selection. In this context, service selection methods have started being adopted for cloud computing in recent years. Therefore, this section provides an overview of the available service selection approaches, and some solutions based on Vehicular Clouds [13,14] and UAV Clouds [15,16].

### 2.1. Service Selection in Vehicular Clouds

A Vehicular Ad hoc Network (VANET) is a network where different vehicles and other devices exchange information using a wireless medium. Given the extensive services and data in vehicular environments, more data processing power is often required than a single vehicle can offer, which may be solved by taking computation to some cloud [17]. The authors in [13] have proposed a new protocol based on LTEA (Long Term Evolution-Advanced) called RCS-VC (Renting out and Consuming Services in Vehicular Clouds) that allows vehicles to rent out their various services while on the move. Moreover, RCS-VC enables user vehicles to discover provider vehicles offering the desired services, and thus consume them. LTEA work as cloud directories, whereby provider vehicles register their offered services, and from which consumer vehicles discover these services.

A new protocol called Discovering and Consuming Cloud Services in Vehicular Clouds (DCCSVC) [18] has been designed to exploit a public bus to rent out services in vehicular clouds. The public buses offer cloud directories for the discovery of supplier vehicle services. Moreover, the same authors propose a new Grid-based Tracking Cell (GTC) technique that allows splitting the predetermined path of each group of buses into several cells grouped in a tracking bus path (TBP), where each TBP has a unique identification (TBPI). Thereafter, when a Provider Vehicle (PV) registers a service at a public bus, it will be informed by the corresponding TBPI. In addition, the fuzzy logic method has been applied in DCCSVC to allow each public bus to select the most suitable supplier.

Tamani et al. [19] proposed Fuzzy Quantified Service Selection (FQSS) for service provider selection within a Mobile Vehicular Cloud. FQSS is a flexible approach based on fuzzy quantifiers and linguistic quantified propositions to aggregate both service constraints and user preferences in polynomial time. The ranking was refined by defining two unique operators: least satisfactory proportion (LSP), and greatest satisfactory proportion (GSP). A Game-theoretic Approach for Service Selection in the Vehicular Cloud (GSS-VC) was proposed in [20]. The objective of this approach is to select the optimal provider vehicles to meet the QoS of consumer vehicles using a cooperative game as a type. The authors selected two players: the consumer vehicle and the provider vehicle. The consumer vehicle can select either Consume (C) or Do Not Consume (NC) options. However, the provider’s vehicle can choose between either Offer (O) or Not Offer (NO). GSS-VC is based on V2V the architecture, since V2I is not taken into account.

The authors in [14] proposed a game theory concept coupled with a quality-of-experience (QoE)-awareness system model to provide drivers with provisioning services in a vehicular environment with low latency, minimal cost, and minimal information to the driver. QoE-awareness collects requirements and preferences, and then assigns a QoE value to every service provider for different types of users; in particular, a framework Multi-Agent/Multi-Objective Interaction Game System (MIGS) has been proposed for providing services in vehicular clouds on demand.

### 2.2. Services Selection in UAV Cloud

In this context, a few approaches for service selection based on UAV Clouds were presented [15,16]. Motlagh et al. [15] proposed two optimisation problems using a Linear Integer Problem (LIP) to select the optimal set of UAVs: (i) Energy Aware Selection of UAVs (EAS), which aims to minimise energy consumption as much as possible, and (ii) Delay Aware Selection of UAVs (DAS), which aims to minimise UAV response times as much as possible. DAS should be applied if the operation time is the target. On the other hand, EAS should be used to minimise energy consumption.

In the UAV resources and services context, the authors in [21] discuss the application of small UAVs in the agriculture field, for object detection, but they faced some issues related to the significant energy consumed when using a recent DL algorithm. A new platform was proposed in [22] that provides a relaying UAV-Cloud by employing the Resource Oriented Architecture (ROA) concept so as to offer UAV resources and capabilities to other requestors via Application Programming Interfaces (APIs). ROA is a client–server architecture based on the Representational State Transfer (REST) architecture. Then, for handling and maintaining dispersed UAV resources, it uses the broker architecture pattern for applications to be more efficient and scalable. This concept is implemented by utilizing the paradigm of Cloud Computing (CC). Moreover, Integrating UAVs with the cloud enables ubiquitous access to UAVs as cloud resources. Furthermore, the UAV-cloud architecture can be seen in relation with providing services. Djeradi et al. [23] proposed a new cloud architecture called universal UAV-cloud architecture (UUCA) comprising three layers: middleware, end-user, and infrastructure. The platform allows a UAV to offer its services to consumers by integrating a new module onboard the UAV called the cloud module. It allows users to request delay-tolerant and real-time UAV wider services. The authors of [23] introduced two scenarios of mobile and fixed users to handle the services offered, along with the overall consumption.

Bousbaa et al. [16] introduced a new game theoretic strategy for service selection in UAV Clouds (GTSSUC) based on the Nash equilibrium interactions between two players: the service provider (UAV), and the requester (client), allowing the customer to select the best service provider in terms of quality and pricing. The interaction aims to select the best strategy to maximize their gains, and where the game’s balance is the condition through which no players can increase their winnings by changing their strategy.

### 2.3. Edge Computing for UAV Networks

Owing to the low computational capability and limited battery capacity of a UAV, multi-access edge computing (MEC) is applied to enhance the performance and mitigate the issues faced by these resource-constrained mobile devices. Several authors [24] have highlighted the possible responsibilities of MEC server integration with UAVs, which is one of the most essential aspects of creating a UAV-MEC system to enhance network performance significantly.

Cao et al. [25] designed a new UAV-MEC system scenario where a UAV offloads its computation tasks to multiple base stations that act as MEC servers. The goal is to minimize the UAV’s mission completion time by optimizing its trajectory concurrently with the computation offloading schedule. The authors in [26] propose a multi-coalition-based UAV MEC network based on the Stackelberg game. Their goal was to optimize computation offloading, the UAV role, and the location selection; their solution showed a high performance in terms of transmission time and energy consumption.

In [27], the authors presented a new generic approach for the computation offloading problem using a UAV-MEC environment. To complete computationally complex operations in a short period of time, they rely on a decentralized system whereby each drone makes the compute offloading decision locally. In particular, it relies on a non-cooperative game to achieve the optimal trade-off between energy consumption, delay, and computation cost. As in [28], the authors propose a coalition-based UAV MEC network using the Stackelberg game, where the UAVs heads act as leaders, and UAVs member act as followers, for the purpose of energy minimization.

In the context of UAV-enabled mobile edge computing, Sahraoui et al. [29] studied the ML-based strategy in UAVs acting as MECs, which collect data using onboard thermal cameras, and then send them to a cloud server for further processing and analysis; this approach allows leveraging ground vehicles as support nodes in the offloading process, and as a power supply source.

Masuduzzaman et al. [30] proposed a UAV-based MEC-assisted system by endowing the UAV with an offloading data processing plan to the MEC server, thereby enhancing the performance of automated traffic management. Additionally, the blockchain technology is introduced to store and secure the traffic record; this way, it provides network repudiation, and avoids any third-party interference within the network.

Furthermore, some works in UAV-MEC environments have involved different approaches, as in [28], where the authors propose a coalition-based UAV MEC network using a Stackelberg game. In their approach, the UAVs heads act as leaders, and UAV members act as followers, and they investigate the computation offloading optimization of UAVs in different layers by combining the channel allocation and position scheduling as well for the purpose of energy minimization. However, unlike our work, this approach did not address the challenge of service selection in terms of price and QoS metric.

Overall, all previous works detailed in this subsection focus on UAV-MEC environments, but fail to address the service selection problem. To the best of our knowledge, no previous work specifically addressed the topic of edge computing for UAV service selection.

## 3. System Model

**Figure 1:**
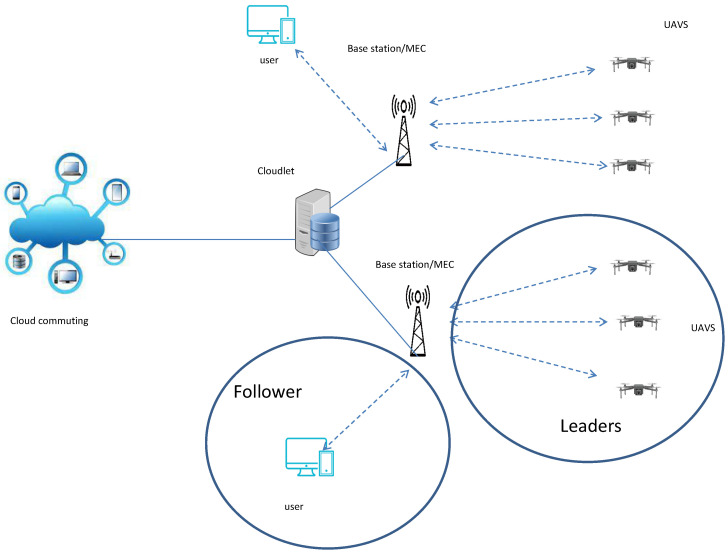
System architecture.

In this section, we start by describing the network model. Figure 1 depicts the envisioned network architecture with three components as follows:

- UAV service provider: entity responsible for making UAV services available to users or consumers.
- User: a requester or client, being the entity responsible for requesting and consuming UAV services.
- BS/MEC server: communication bridge between the user and the provider. It can be an entity for storing all offers from UAV service providers so as to be consumed by the user.

As illustrated in Figure 1, UAVs can offer their services via a BS, and act as a MEC server node near users, providing low latency, reduced bandwidth requirements, and improved security. In general, users and UAV service-providers exchange prices and demand information through the BS/MEC server. All providers publish the price of their services, as well as all the relevant information about energy and delays. Thus, the users transmit their demands to the UAV service-provider based on the cost of the service and its availability, while also considering the available budget for the requested service. Therefore, it is challenging for each user to choose the best overall service.

Table 1 gives a summary of the key definitions and abbreviations used in this work.

## 4. Problem Formulation

**Figure 2:**
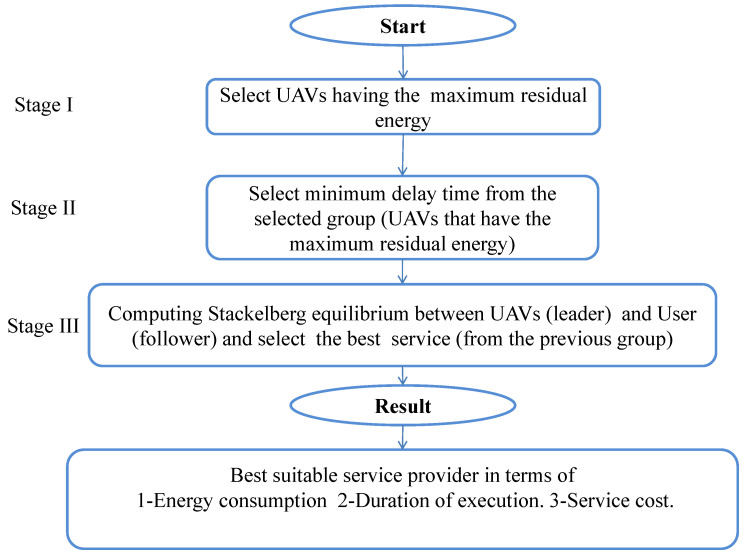
Proposed architecture for selecting appropriate UAVs for MEC service provision.

As depicted below in  Figure 2, that presents the structure diagram for selecting appropriate UAVs, we have three stages in our work. Stages I and II are used for selecting the appropriate UAVs by accounting for the maximum residual energy and the minimum delay time, respectively. Stage III is for computing a Stackelberg game between UAVs (leader) and the users (followers) for selecting the best service. In this approach, each service is represented by a number of data packets.

### 4.1. Selecting the Appropriate UAVs

The residual energy in each selected UAV should be higher than a predefined threshold:

(1) $$E^{Residual}>E^{Threshold}$$

where the time delay does not exceed a predefined threshold either:

(2) $$DT>DT^{Threshold}$$

Algorithm 1 illustrates how to select suitable UAVs in terms of residual energy and delay time.
**Algorithm 1** Selection of suitable UAVs.**Input Value:**Us: set of all UAVsx: UAVEth: threshold residual energyDTth: threshold delay time**Output:**Ud: set of UAVs have minimum delay time from the selected group (Ue)Er(x): residual energy of UAV x,DT(x): Delay time of UAV x**Begin**  1:Ud←∈ϕ;  2:**for** each x∈Us **do**  3:      **if** $\left(Er\left(x\right)>E^{th}\right)\&\left(Dt\left(x\right)>Dt^{th}\right)$ **then**  4:            $Ud\leftarrow Ud⋃\left\{x\right\}$  5:▹ Compute a “Stackelberg game” Between Ud and User                                                       6:      **end if**  7:**end for**  8:**End**

The complexity of Algorithm 1 depends on the size of the input set of UAVs (Us), and the time required to compute the Stackelberg game in line 4. Hence the time complexity of the algorithm is O(|Us|) if the computation of the Stackelberg game takes a constant time. The size of the output collection Ud determines the space complexity of Algorithm 1. Since Ud is a subset of Us, its size cannot exceed Us’s size, and hence the algorithm’s space complexity is O(|Us|).

### 4.2. Example Scenario: Interaction between UAV and User

In this section, we will look at a straightforward example so as to better demonstrate and provide a clearer understanding of the functionality of our approach. We consider that both the service provider (UAVs) and the user are within the base station’s transmission range. In this scenario, we assume the BS acts as a MEC server that can store all offers sent by a service provider.

**Figure 3:**
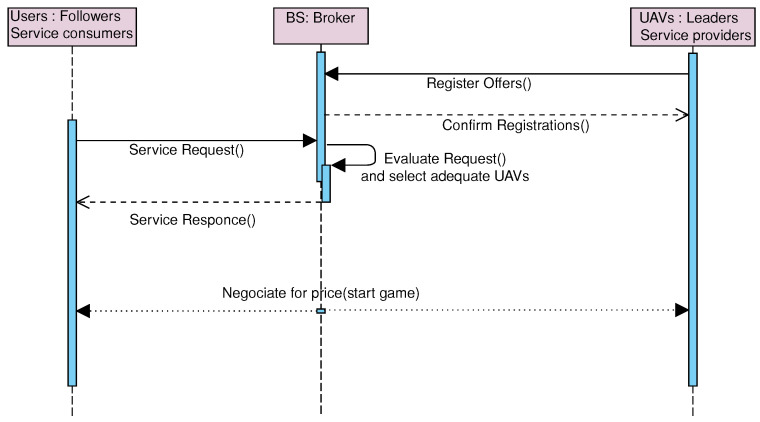
The Stackelberg game approach interaction diagram in a UAV-based MEC.

Figure 3 shows a sequence diagram depicting the interaction in the Stackelberg game scenario between the users (as followers) and UAVs (as leaders). In this context, the base station (BS) receives the service providers’ offers that tend to maximize their global utility, and then, through the users’ requests, the appropriate UAVs that respond to the service requests are selected in terms of remaining energy and delay. The interested customers are notified to start the process of negotiations on the price until reaching a point of agreement that maximizes the price of the UAVs that a customer is willing to pay for a service.

## 5. Stackelberg Game Theory Approach

The Stackelberg game method strives to choose appropriate services in UAV Clouds, allowing the client or requester to select the most suitable service provider.

Game theory (GT) is an area of applied mathematics that looks at how the strategies of multiple people making decisions affect each other (players). These decision-makers work together or against each other to make smart choices that have different results [31].

The Stackelberg model is sometimes called *leader-follower interactions*, and is often used to describe interactions between a dominant player (leader) with other players (followers). Hence, a leader acts first, and followers react afterward to satisfy the leader’s strategy. This model is known as the Stackelberg model in economics [32].

### 5.1. Utility of Service Users (Phase 2)

The users react to the UAVs service provider according to the price of the requested service. The utility of each user is represented by the Ui, being formulated as follows:

(3) $$\begin{array}{c} \max_{qi,j}\;ui=Bi\sum_{i\in Ud}\log\left(\alpha i+qij\right)\\ \;\mathrm{St}\;\sum_{j\in Ud}Pjqij\leq Bi\\ qij\geq o\forall j\in Ud \end{array}$$

The log function has been utilized extensively in network optimization problems, and used to determine Fisher market equilibrium allocations [33]. Here, αi is a constant that is usually equal to 1, since Bi and qij represent the budget and the number of services obtained by the consumer from the provider, respectively. The demand strategy (inspired by studies [34,35]) is formulated as follows:

(4) $$qij=\left\{\begin{array}{c} \frac{Bi+\alpha i\sum_{j=1}^{U_{d}}p_{j}}{Npj}-\alpha_{i}\;\;\leftarrow q_{ij}\geq 0\\ 0\leftarrow q_{ij}<0 \end{array}\right.$$

If the UAVs’ price service ($p_{j}$) is excessively high compared to the budget Bi, several $q_{ij}$ values may be negative. This contradicts the requirement that no USER will have a negative demand. In this case, $q_{ij}$ will be 0 instead.

Algorithm 2 computes the optimal service request depending on the price published by the UAV provider service.
**Algorithm 2** Determine best demand strategy.**Input Value:**$U_{d}$: a set of appropriate UAVs.$\alpha_{i}$: constant.Bi: budget of User${}_{i}$ for a service.$p_{j}$: the current price of service of jth Ud.**Output Value:**$q_{ij}$: optimal demand for a service.**Begin**  1:sumP ← sum of current price of each UAVs ∈$U_{d}$  2:**for** each User${}_{i}$ **do**  3:    **for** each $U_{dj}$ **do**  4:        $q_{ij}\leftarrow 0$  5:        **if** $p_{j}\neq 0$ **then**  6:           $q_{ij}\leftarrow \frac{Bi+\alpha i\cdot \mathrm{sumP}}{N\cdot p_{j}}-\alpha_{i}$  7:           **if** $q_{ij}$ < 0 **then**  8:               **set** $q_{ij}\leftarrow 0$  9:           **end if** 10:        **end if** 11:    **end for** 12:    **end for** 13:    **return** $q_{ij}$**End**

The algorithm computes the optimal demand for service $q_{ij}$ for each user based on a set of parameters. In particular, it iterates over each user, and then calculates its value according to the formula mentioned above (4) based on the value of the current price $p_{j}$, and the sum of the current services price of all UAVs in a set $Ud_{j}$.

The time complexity of this algorithm depends on the first loop, which iterates for all users (*m* times), and the second loop, which iterates for $Ud_{j}$ (*n* times), thus resulting in a time complexity of O(n). Therefore, the overall time complexity of the algorithm can be expressed as O(m×n)

Overall, the space complexity of the algorithm is determined by the amount of memory required to store the input, output, and any intermediate variables used by the algorithm. In this case, the only variables used by the algorithm are the input variables and the output variables. Therefore, the space complexity of the algorithm is O(n+m).

### 5.2. Utility of Service UAVs (Ud) (Phase 1)

The UAVs initiate the game by announcing the price of each service. Afterward, the follower has to choose among different demand strategies using the service’s price provided. Presumably, the leader UAVs are aware their actions affect the demand choices of follower users (Algorithm 2). If a certain UAV${}_{j}$ sells its services at a greater price than other UAVs, then users are more likely to purchase services from UAVs with lower prices. This occurs because the interactions between UAVs are competitive interactions. As a result, leader UAVs should know of their effect on follower consumers when setting prices for their services. Thus, to maximize utility, UAVs should choose suitable price for their services according to the following equation:

(5) $$\begin{array}{c} \max_{P_{j}}\;U_{{ud}_{j}}\left(p_{j},p_{∼}j\right)=p_{j}qij\\ \;\mathrm{St}.\;\sum_{j\in C}q_{ij}\leq G_{j}\\ where\;\;\;\;q_{ij}=\left\{\begin{array}{c} \frac{Bi+\alpha i\sum_{j=1}^{N}p_{j}}{Npj}-\alpha i\\ 0 \end{array}\right.\\ p_{j}\geq 0\;\;\forall j\in Ud \end{array}$$

where $p_{∼}j$ is the price vector of service for all UAVs except *UAV*${}_{j}$; hence, given $p_{∼}j$, *UAV*${}_{j}$ will choose an optimal price vector $p_{j}$.

### 5.3. Game Formulation and Equilibrium Analysis

In this section, we model the interaction between the UAVs and the users as a Stackelberg game.

In the first phase of the game, UAVs choose and publish their strategies. This means we can apply the concept of Nash equilibrium (NE) among UAVs to determine the service price.

In the second phase, users will decide their demands based on their budgets, and the price offered by the service provider. Through Algorithm 2, users can obtain the optimal service request considering that:

- Players: Users and UAVs.
- Strategies: Each $UAV_{j}$ selects its service price $p_{j}$, whereas users choose the optimal demand.

This way, we obtain p* and q* to reach a Stackelberg equilibrium (SE) of the game between UAVs and users, where (p*, q*) is a SE for the proposed Stackelberg game for any (*p*, *q*), with *p* > 0 and *q* > 0, and satisfying the following conditions:

(6) $$\begin{array}{c} U_{{ud}_{j}}\left(p*,q*\right)\geq U_{{ud}_{j}}\left(p_{j};p_{∼}^{*}j,q\right),\;\;\forall j\in Ud, \end{array}$$

(7) $$\begin{array}{c} Ui\left(p*,q*\right)\geq Ui\left(p^{*},q_{i};q_{∼i}^{*}\right),\;\;\forall i\in C. \end{array}$$

In the initial phase of this Stackelberg game, UAVs will choose price strategies and reveal those choices to the users. We consider the Nash equilibrium as a solution for determining prices among UAVs. In the second phase, users will take into account both their budgets and the service price offered by each UAV before determining their service needs. The suggested algorithm (Algorithm 2) allows users to achieve the best possible demand. Thus, the suggested Stackelberg game has a unique Stackelberg equilibrium, which is presented as Algorithm 3.
**Algorithm 3** Stackelberg Equilibrium.  1:**for** each iteration *r* **do**  2:UAVs advertise the initial price of service to users  3:Users compute the optimal demand (Algorithm 2) depending on the published price (Ud)  4:    **for** each $UAV_{j}\in$ Ud **do**  5:        **if** $\sum_{i=1}^{C}q_{ij}<G_{j}$ **then**     Decrease the service price  6:                $NewPrice=OldPrice+(\sum_{i=1}^{C}q_{ij}-G_{j})\times \lambda$  7:        **else**  8:           **if** $\sum_{i=1}^{C}q_{ij}>G_{j}$ **then**     Increase the service price  9:                    $NewPrice=OldPrice+(\sum_{i=1}^{C}q_{ij}-G_{j})\times \lambda$ 10:           **end if** 11:        **end if** 12:        **if** ∣NewPrice−OldPrice∣<ϵ **then** 13:           $p_{j}^{*}\leftarrow NewPrice$ 14:           **break** 15:        **end if** 16:    **end for** 17:    r←r+1 18:**end for**

This algorithm is a basic implementation of the Stackelberg equilibrium of a price adjustment mechanism that aims to find the optimal price for UAV services. It operates in iterations, with each iteration denoted by the variable *r*. The process can be summarized as follows:

1. In each iteration, UAVs publish the initial price of their service to users.
2. Users then compute the optimal demand of service they would like to receive based on the published prices by the UAVs (using Algorithm 2).
3. For each $UAV_{j}$ in the set of Ud, the algorithm performs the following steps:
  - If the optimal service demand for all users is less than the total number of available services of $Ud_{j}$ (denoted by Gj), the service price decreases.
  - If the optimal service demand for all users is greater than the total number of available services of $Ud_{j}$ (denoted by Gj), the price of the service is increased.
4. The algorithm checks if the difference between the NewPrice and the OldPrice is smaller than a specified tolerance. If this condition is satisfied, the NewPrice is considered as the final price for $UAV_{j}$, and the algorithm breaks out of the loop for that UAV.
5. The loop for each $UAV_{j}$ in the Ud set continues until the condition in step 4 is satisfied.

The complexity of this algorithm depends on various factors, including the set size for Ud, the number of iterations performed, and the computational complexity of Algorithm 2, O(n×m), which is used to compute the optimal demand of service. Therefore the overall time complexity can be estimated as O(r(n+n×m)). This means that the algorithm’s running time grows linearly with the number of Ud and the number of iterations.

As final remarks, notice that we need to store the demand values for each user/UAV combination. Additionally, we must store each UAV’s prices, contributing to the space complexity. Overall, the space complexity is O(nm+n). This indicates that the space complexity of the algorithm also increases linearly with the number of UAVs and iterations performed, as well as with the size of the input data and the intermediate results.

## 6. Performance Evaluation

To analyze our proposed solution, we carried out our experiment using the NS-3 simulator. The first scenario adopted consists of four UAVs that move according to a Gauss–Markov process in a 3D environment at a random speed in the interval [80, 120], with three users, leveraging one base station as a broker, and then varying either the number of UAVs and users, as required to speed up the algorithm’s convergence. We set the accuracy requirement ϵ = 1 × 10${}^{-10}$, and the step parameter λ = 0.05. Table 2 summarizes the simulation parameters.

Our simulation’s performance metrics are listed below.

- Successful Execution Ratio: A measure of the success rate of a process, task, or operation. It represents the ratio that a process is successfully completed relative to the total number of execution attempts.
- Service Utility: Represents client gains after using services, and refers to a consumer’s satisfaction or usefulness for a particular service. In other words, it is the value that a service provides to a customer.
- Average response time: Represents the total time from the instant a request is sent to the base station until the reception of the last data packet.
- Throughput: Refers to the maximum amount of data that the receiver receives from the sender when transmitted over a network within a given period of time.
- Average residual energy of the network: Illustrates the remaining energy available in the network nodes after they have been used for communication and computation.

**Figure 4:**
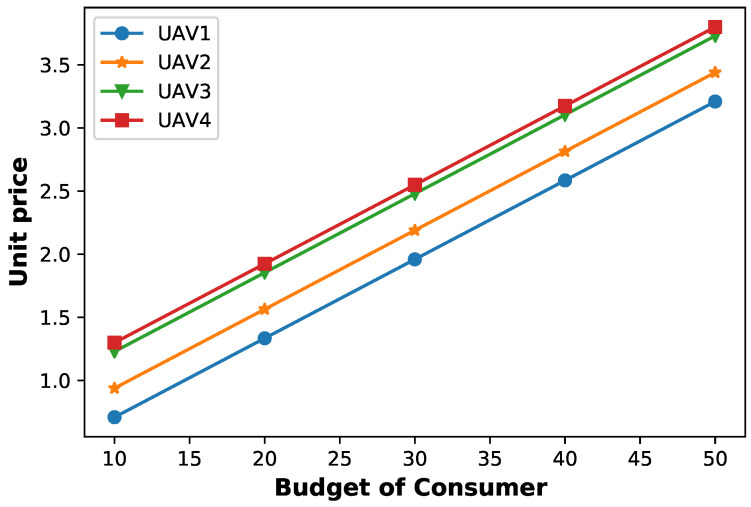
Optimal UAV service prices at the Stackelberg equilibrium when varying the consumer budget.

Figure 4 shows the optimal service rates offered by each UAV under Stackelberg equilibrium. Notice that, when user 1’s budget for service consumption varies from 10 to 50, we can observe that increases in service unit price are directly proportional to the increase in the users’ budget; this is required in order to maximize its own utility.

**Figure 5:**
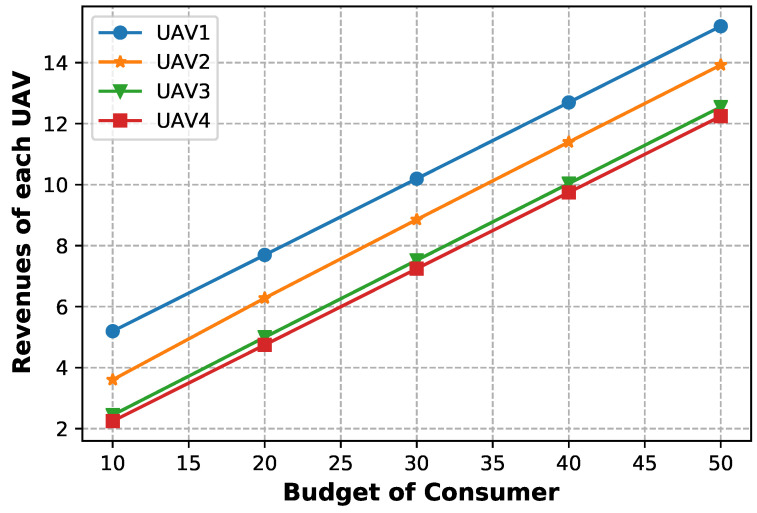
Revenues of UAVs at the Stackelberg equilibrium when varying the consumer budget.

Figure 5 shows the revenue of each drone when the total budget of User 1 varies from 10 to 50. Although UAV 1 has the lowest unit price, the corresponding revenue is the highest because service consumers tend to demand affordable services.

**Figure 6:**
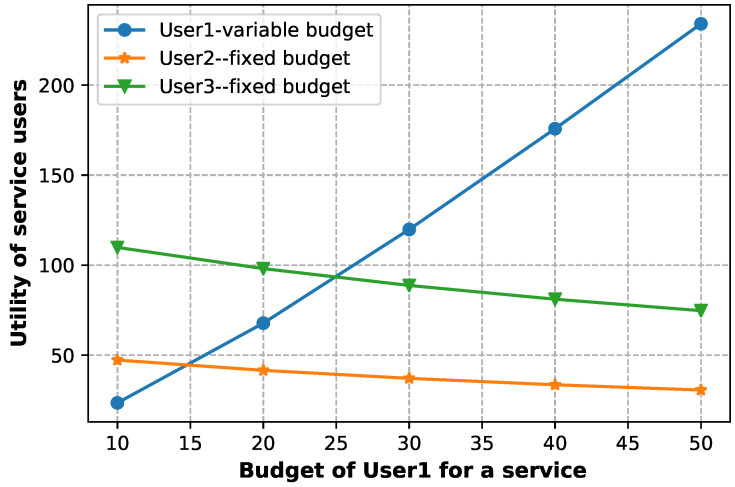
Utilities of service users at the Stackelberg equilibrium when varying the budget of user 1.

Figure 6 shows each user’s benefit under Stackelberg equilibrium. The benefit of User 1 increases with the increase in his budget, while the benefit of other users (who have a fixed budget) decreases due to the increase in the price of the service, which was caused by the increase in the budget of User 1.

**Figure 7:**
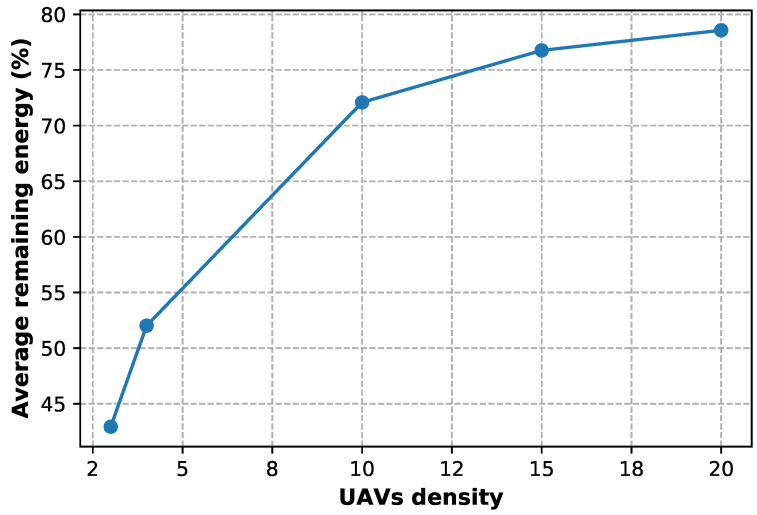
Average remaining energy according to the UAVs density.

To avoid having the remaining battery lifetime of UAVs becoming very low, which leads to a sudden service interruption for the user, the broker (base station) selects only the UAVs that respond to the criteria previously set by the users’ request in terms of energy and delay. Figure 7 shows that the average remaining energy of UAVs increases as the UAV density increases in the Stackelberg game, which means decreased competition for service demand, and so users have more choices to consume.

**Figure 8:**
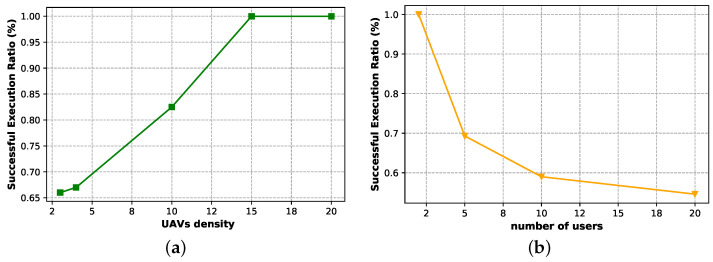
The successful execution ratio when varying (**a**) the UAV density, and (**b**) the users density.

Figure 8a shows the increasing rate of the successful execution ratio when varying the UAV density in our Stackelberg game; the achieved behaviour is expected due to the availability of additional UAVs, which enables more services to be offered to the consumers until the maximum is reached. On the contrary, an increase in the number of users leads to the reverse process, as depicted in Figure 8b. In this case, the successful execution ratio decreases because there are some users who request the service from some UAVs, which in turn are serving other consumers, or the users failed to choose the best service, which in turn will lead to reducing the service gain.

**Figure 9:**
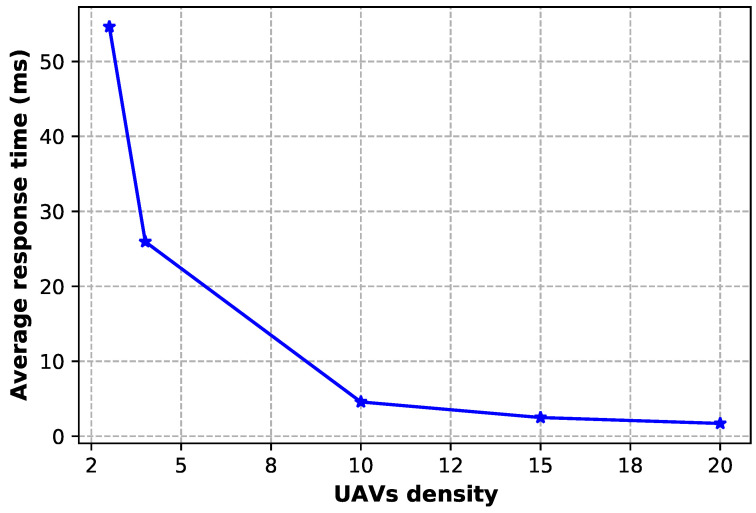
Average response time when varying the UAV density.

From Figure 9 we can observe that the response time is inversely proportional to the UAV density; thus, the greater the number of UAVs, the lower the response time. This is due to the presence of additional resources provided by other UAVs, which enables avoiding competition for limited resources, thereby decreasing response time.

**Figure 10:**
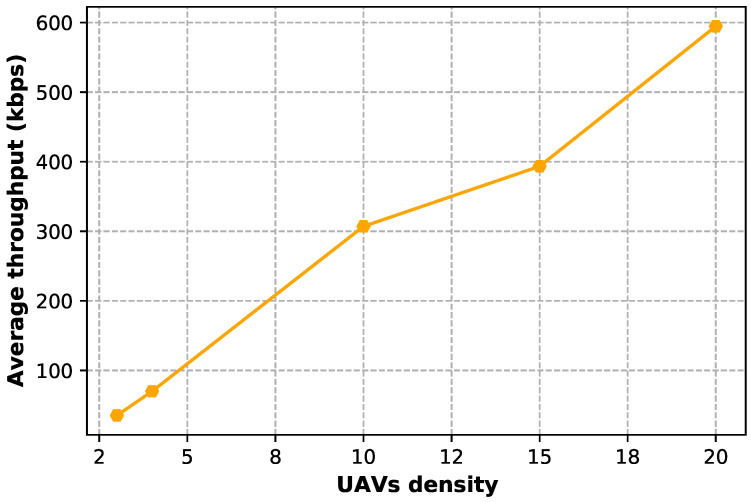
Average throughput when varying the UAV density.

Figure 10 shows, for each consumer, the average throughput when varying the UAV density. It can be observed that, if a higher number of UAVs are present in the Stackelberg game scenario, the average amount of data exchanged per user increase due to an increase in the availability of UAV resources, hence allowing for more services to be provided to consumers.

## 7. Conclusions

In this paper, we investigated a game theoretic approach to service selection in the UAV cloud. In particular, we propose a Stackelberg game whereby users act as followers, and UAVs act as leaders. Such approach allows users (or requesters) to select the best service and a suitable price, while also enabling the service providers (UAVs) to choose the most optimal strategies to maximize utility.

Due to the massive amount of data UAVs can collect, the selection process becomes quite complex. For this reason, the results of this paper contribute to an efficient service-selection process in UAV clouds, allowing clients to efficiently determine the most appropriate provider, while considering the consumer’s preferences and the constraints imposed by the service.

The results obtained using the NS-3 simulator demonstrate that the proposed game is efficient in terms of UAV’s revenue and QoS metrics.

As future work, we will attempt to detect insider attackers, whereby the attacker can enter the game either as a leader or a follower, to reduce the benefits of both parties. Furthermore, since the secure service selection process in the UAVs cloud is an important research area to be investigated, we will propose a secure game by implementing blockchain technology between leaders and followers.

## Figures and Tables

**Table 1:** Summary of key definitions.

Notation	Definition
Bi	Budget of user i for a service
pj	Service price of provider j
qij	number of service obtained by user i from UavServiceProvider j
Gj	Total number of available services in UavServiceProvider j
Us	set of all UAVs
Ue	set of all Us which have max residual energy
Ud	set of all Ue which have minimum delay time
C	set of users
$E^{Residual}$	residual energy
$E^{Threshold}$	threshold residual energy
Dt	Delay time
$Dt^{Threshold}$	threshold delay time
N	Total number of UAV service providers

**Table 2:** Simulation parameters.

Parameter	Value
Simulation time	50 s
UAV density	3, 5, 10, 15, 20
Number of users (consumers)	1, 2, 5, 10, 20
UAV speed	[80, 120] km/h
Mobility model	Gauss–Markov 3D
Propagation loss model	Nakagami-m
ϵ	1 × 10${}^{-10}$
λ	0.05

## Data Availability

All implementation details, sources, and data will be delivered upon requesting the corresponding author Carlos T. Calafate.
